# Transmission of alpha-synuclein in Parkinson’s disease

**DOI:** 10.1186/1750-1326-8-S1-O25

**Published:** 2013-09-13

**Authors:** Virginia M-Y Lee

**Affiliations:** 1 University of Pennsylvania, USA

## 

The accumulation of misfolded proteins is a fundamental pathogenic process in neurodegenerative diseases. However, the factors that trigger aggregation and spreading of α-synuclein (α-syn), the principal component of the intraneuronal inclusions known as Lewy bodies (LBs) and Lewy neurites (LNs) that characterize Parkinson’s disease (PD) and dementia with LBs (DLB) in brain are poorly understood. We demonstrate that pre-formed fibrils (pffs) generated from recombinant α-syn enter cultured primary neurons generated from non-transgenic wildtype mice, promote recruitment of soluble endogenous α-syn into insoluble PD-like LBs and LNs. Accumulation of pathologic α-syn led to selective decreases in synaptic proteins, progressive impairments in neuronal excitability and connectivity, and eventually, neuron death. Moreover, we further show that in young asymptomatic α-syn transgenic (Tg) mice, intracerebral injections of synthetic α-syn pffs accelerate both the formation of intracellular LB/LN-like inclusions and the onset of neurological symptoms in recipient animals. Pathologic α-syn propagated along major central nervous system (CNS) pathways to regions far beyond injection sites and dramatically reduced survival with a highly reproducible interval from injection to death in inoculated animals. Thus, synthetic α-Syn pffs are wholly sufficient to initiate PD-like LBs/LNs and to transmit disease in primary neurons *in vitro* and Tg mice *in vivo.* Thus, these data support a prion-like cascade in synucleinopathies whereby cell-to-cell transmission and propagation of misfolded α-syn underlie the CNS proliferation of LBs/LNs. These findings open up new avenues for understanding the progression of PD and for developing novel therapeutics.

